# Comparing human exposure to fine particulate matter in low and high-income countries: A systematic review of studies measuring personal PM_2.5_ exposure

**DOI:** 10.1016/j.scitotenv.2022.155207

**Published:** 2022-04-11

**Authors:** Shanon Lim, Eridiong Bassey, Brendan Bos, Liberty Makacha, Diana Varaden, Raphael E. Arku, Jill Baumgartner, Michael Brauer, Majid Ezzati, Frank J. Kelly, Benjamin Barratt

**Affiliations:** a MRC Centre for Environment and Health, Imperial College London, UK; b NIHR-HPRU Environmental Exposures and Health, School of Public Health, Imperial College London, UK; c Place Alert Labs, Department of Surveying and Geomatics, Faculty of Science and Technology, Midlands State University, Zimbabwe; d Department of Women and Children’s Health, School of Life Course Sciences, Faculty of Life Sciences and Medicine, King’s College London, UK; e Department of Environmental Health Sciences, School of Public Health and Health Sciences, University of Massachusetts, Amherst, USA; f Institute for Health and Social Policy, and Department of Epidemiology, Biostatistics and Occupational Health, McGill University, Montreal, Canada; g School of Population and Public Health, The University of British Columbia, Vancouver, Canada; h Institute for Health Metrics and Evaluation, University of Washington, Seattle, USA; i Abdul Latif Jameel Institute for Disease and Emergency Analytics, Imperial College London, UK; j Regional Institute for Population Studies, University of Ghana, Legon, Ghana

**Keywords:** Personal exposure, Fine particulate matter, High-income, Low-income, Rural, Urban

## Abstract

**Background:**

Due to the adverse health effects of air pollution, researchers have advocated for personal exposure measurements whereby individuals carry portable monitors in order to better characterise and understand the sources of people’s pollution exposure.

**Objectives:**

The aim of this systematic review is to assess the differences in the magnitude and sources of personal PM_2.5_ exposures experienced between countries at contrasting levels of income.

**Methods:**

This review summarised studies that measured participants personal exposure by carrying a PM_2.5_ monitor throughout their typical day. Personal PM_2.5_ exposures were summarised to indicate the distribution of exposures measured within each country income category (based on low (LIC), lower-middle (LMIC), upper-middle (UMIC), and high (HIC) income countries) and between different groups (i.e. gender, age, urban or rural residents).

**Results:**

From the 2259 search results, there were 140 studies that met our criteria. Overall, personal PM_2.5_ exposures in HICs were lower compared to other countries, with UMICs exposures being slightly lower than exposures measured in LMICs or LICs. 34% of measured groups in HICs reported below the ambient World Health Organisation 24-h PM_2.5_ guideline of 15 μg/m^3^, compared to only 1% of UMICs and 0% of LMICs and LICs. There was no difference between rural and urban participant exposures in HICs, but there were noticeably higher exposures recorded in rural areas compared to urban areas in non-HICs, due to significant household sources of PM_2.5_ in rural locations. In HICs, studies reported that secondhand smoke, ambient pollution infiltrating indoors, and traffic emissions were the dominant contributors to personal exposures. While, in non-HICs, household cooking and heating with biomass and coal were reported as the most important sources.

**Conclusion:**

This review revealed a growing literature of personal PM_2.5_ exposure studies, which highlighted a large variability in exposures recorded and severe inequalities in geographical and social population subgroups.

## Graphical abstract

**Graphical Abstract:**
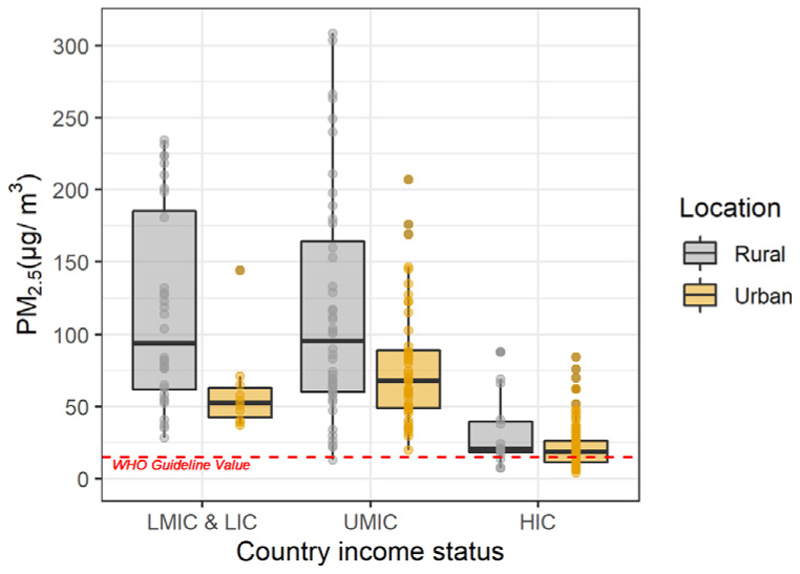

## 1 Introduction

The detrimental impact of air pollution on health is well established with an estimated 7 million premature deaths per year attributable to household and ambient air pollution (World Health Organisation, 2021a). These health outcomes have typically been estimated for populations based upon outdoor air pollution concentrations measured at central fixed monitors (Brokamp et al., 2019), using satellite imagery and dispersion models to predict concentrations outside people’s homes (de Hoogh et al., 2018) or by combining estimates of the proportion of households that cook primarily with solid fuels as proxy for indoor air pollution (Health Effects Institute, 2020). These approaches are often implemented as cost-effective methods to estimate exposures for large populations, as they are important for assessing the general health effects of air pollution. However while these estimates are predictive of population health responses, they may not accurately represent the air pollution that specific individuals breathe (Health Effects Institute, 2010; Smith, 1993).

Personal monitoring whereby participants wear or carry portable air pollution monitors is considered the ‘gold standard’ for individual air pollution exposure assessment (Brauer et al., 2002; Brokamp et al., 2019; Clark et al., 2013; Health Effects Institute, 2010). Personal exposure measurements have often been utilised to better understand the characteristics of the population’s exposure, particularly as exposure varies by age, occupation, location, and activity (Snyder et al., 2013; Steinle et al., 2013). In recent years, the increased availability of compact monitoring technology has stimulated new personal exposure research focussing on portable, high time resolution instruments (often paired with GPS devices) that can be used to measure the spatial and temporal variability in personal exposure in people’s daily lives (Snyder et al., 2013). Portable high resolution air pollution monitors can therefore provide a better understanding of the various sources and levels of air pollution an individual is likely to be exposed to throughout the day (Steinle et al., 2013) compared to the traditional gravimetric time-integrated monitors. By providing a detailed characterisation of people’s exposure, targeted interventions which have the greatest impact in reducing exposures can be formulated. This can be particularly important for vulnerable sub-populations.

There have been reviews focussing on pollutant concentrations in transport and indoor microenvironments which highlighted that undertaking active travel reduces exposure to pollutants compared to motorised transport (Cepeda et al., 2017; de Nazelle et al., 2017; Karanasiou et al., 2014) and the importance of indoor activities such as cooking, heating and smoking in the contribution to total personal exposure (Morawska et al., 2013; Vardoulakis et al., 2020). Other reviews have assessed the use of ambient monitors to represent personal exposure and found that the participants and environments of studies affect the accuracy of using ambient monitors as a proxy and typically do not represent personal exposure levels (Avery et al., 2010; Evangelopoulos et al., 2020). However, the majority of these reviews have only included studies in Europe or North America (Avery et al., 2010; Evangelopoulos et al., 2020) and often explicitly exclude lower income countries (Karanasiou et al., 2014; Morawska et al., 2013). More recently, there have been reviews on lower income countries that have focussed on air pollution exposure and concentrations in transportation (Kumar et al., 2018) and household air pollution from cookstoves (Carter et al., 2017; Clark et al., 2013; Quansah et al., 2017; Thomas et al., 2015). These reviews highlighted that household air pollution in these countries often exceed air quality guideline levels and that current stove interventions only provide small reductions in exposure. Despite studies advocating for the measurement of personal exposures, no review has been conducted on the global characteristics and magnitude of personal particulate matter exposure studies to date.

Fine particulate matter, defined as particles less than 2.5 μm in diameter (PM_2.5_), is a frequently studied pollutant due to its wide range of identified adverse health effects. Ninety percent of the world’s population is now thought to live in areas where ambient PM_2.5_ concentrations are above the 2005/interim target 4 World Health Organisation’s (WHO) annual air quality guideline of 10 μg/m^3^ (Shaddick et al., 2020; World Health Organisation, 2021b). Reducing PM_2.5_ concentrations has been challenging, in part due to the wide range of sources such as cooking, heating, cigarette smoke, household cleaning, emissions from vehicles, wind-blown dust, and industry (Karagulian et al., 2015; Vardoulakis et al., 2020).

There is also a severe inequality in relation to the health outcomes of air pollution with 94% of ambient and household PM_2.5_ related deaths estimated to occur in low and middle income countries, at a three times higher rate per capita compared to high income countries (World Health Organisation, 2018). However, despite the known inequality on the health effects of air pollution (Health Effects Institute, 2020), there is increasing awareness that effective policies which may mitigate air pollution exposure in high income countries may not be applicable in other areas. Undertaking personal exposure monitoring of sub-populations can assist in identifying the determinants of their exposure and therefore can provide targeted interventions to reduce this exposure.

This review builds on a number of themes highlighted from the early 1990s (Smith, 1993, 2002), such as the need for better exposure assessment in air pollution studies, the importance of quantifying microenvironment exposure particularly indoors and the inequality of pollution exposure in developing countries. However, historically portable monitoring technology was not available to conduct accurate, time-resolved personal exposure assessments and agreement over which pollutant and the size of particles to measure was not well established.

The primary aim of this systematic review is to assess the differences in the magnitude and sources of personal PM_2.5_ exposures experienced between countries at contrasting levels of income. Additionally, we compared personal exposures by gender, age, and urban and rural locations. The review is structured by first detailing how studies were selected and how personal PM_2.5_ exposure measurements were summarised. The results present the study locations, groups monitored and outline the magnitude of PM_2.5_ exposures measured across countries at different levels of income. This is followed by further detail on the sources of exposures and their magnitude in high (HIC), upper-middle (UMIC), lower-middle (LMIC), and low (LIC) income countries. The outcome of this review identifies gaps in the current literature and recommends future directions of research to advance personal PM_2.5_ exposure studies.

In this manuscript, we use the word ‘exposure’ to mean pollution levels experienced by individuals, whereas ‘concentration’ is defined as pollutant levels in a particular location measured by a fixed monitor, indoor or outdoor.

## 2 Methods

### 2.1 Search strategy

This systematic review was guided by the Preferred Reporting Items for Systematic Reviews and Meta-Analyses (PRISMA) statement (Page et al., 2021). We searched in PubMed and Web of Science databases from 1 January 2000 to 19 August 2020 for papers in English that measured and reported the magnitude of personal PM_2.5_ exposure for a population. The references of identified studies and the global household air pollution database (Shupler et al., 2018) were also reviewed for relevant publications. Our review protocol was not registered as PROSPERO only accepts systematic review registrations with a health-related outcome. Our review protocol and search terms are presented in SI.1.

### 2.2 Study selection

We included any study where personal exposures were directly measured by participants carrying a PM_2.5_ monitor for at least 20 continuous hours throughout their typical day. The 20-h limit was to ensure comparability of daily exposure variations for each studied population ensuring that measurements included a period when the participant was asleep.

Studies with less than 10 unique participants were excluded, as they were deemed to not have a broad enough representation of the targeted population monitored. Studies were also excluded if no original data was analysed, if they measured exposure during specific events (e.g. festivals, pollution control periods, etc.), simulated expected population activities, modelled or assigned personal exposures based on fixed monitors, or estimated personal exposure based on microenvironment concentrations and time-activity data. Studies which modelled PM_2.5_ exposure based on carbon monoxide measurements were also excluded as a previous review suggested that carbon monoxide is not a good proxy measure (Carter et al., 2017).

The study selection was first based on a review of titles and abstract by S.L. and one of E.B., B.Bos, L.M. Papers that were related to or mentioned personal exposure in their title or abstract then had their full text screened to review if they met the inclusion criteria above. Only peer-reviewed published journal articles and reports were included in this review.

### 2.3 Data extraction

Data was extracted using a preestablished template in Microsoft Excel (SI.1). The key outputs for each paper were extracted by one author (S.L.) and included sample size, study location, participant category (i.e. age, gender, occupation, health, smoking status), season, heating/cooking fuel, intervention tested (if applicable), personal monitor type, time resolution of monitoring, quality assurance and control protocols, summary statistics of personal exposure (where available: arithmetic and geometric mean, median, arithmetic and geometric standard deviation, minimum, maximum and interquartile range), summary statistics of ambient concentrations, the correlation between personal exposure and ambient concentration and details of microenvironments where the participants spent time. The measurement methodology was also recorded based on whether studies used time-integrated or time-resolved monitors to measure personal exposure. The outputs were then validated (by one of E.B., B.Bos, L.M.), with any disagreements in outputs resolved through discussion.

### 2.4 Quality assessment

As there is no standard quality assessment criteria for personal air quality exposure studies we implemented a subjective quality rating based on methods used to assess the accuracy of personal monitors and the representativeness of the monitored population. The accuracy of personal monitors was based on the QA/QC protocols reported, good QA/QC was determined for time-resolved monitors by being co-located to gravimetric filter measurements or a reference monitor, while good QA/QC using time-integrated gravimetric monitors was when studies followed standard procedures i.e. blank filter correction, flow calibration, filters processed in a controlled temperature lab, replicate filter weighing (e.g. Shan et al., 2014). The representativeness of the monitored population was solely based on sample size, this was decided at 30 unique participants. This value was decided arbitrarily and was based on balancing the logistics and cost of conducting a personal exposure study, alongside whether the summary PM_2.5_ exposure value would be broadly representative of the population. A study with a sample size less than 30 participants which did not report QA/QC procedures was labelled with a “low” rating, a study with good QA/QC or sample size greater than 30 was labelled as “moderate”, while a study with good QA/QC and sample size greater than 30 participants was labelled as “high”.

### 2.5 Data analysis

Due to the variety of study designs (e.g. averaging times, populations targeted, study duration, and measurement techniques) and range of exposures, a meta-analysis was not performed. Instead, a quantitative synthesis of PM_2.5_ exposures was conducted. Exposures were primarily summarised into LIC, LMIC, UMIC, and HIC (World Bank, 2020) and urban or rural locations (urban was defined as a location with a population greater than 100,000 residents). World Bank (2020) designation places LIC with a gross national income per capita at less than US$1035, LMIC less than US $4045, UMIC less than US$12,535 and HIC greater than US$12,536. Summary personal PM_2.5_ exposure measurements within studies were also split between participant age, gender, occupation, seasons, smoking status, health status, and intervention group (such as participants using indoor air filters or different cooking stoves). Exposures recorded in this review were based on the groups presented (defined as “group measurements”) within each paper, therefore a single study could provide multiple personal PM_2.5_ measurements (e.g. if a study summarised exposure between males and females, two measurements were recorded).

The median, 25th and 75th percentile, minimum and maximum mean study exposures were summarised to provide an indication of the distribution of PM_2.5_ exposures measured within each country income category and by gender, age, rural-urban locations. As approximately 75% of the studies reported arithmetic means (AM), this was chosen as the standardised metric of comparison, and where possible geometric means (GM) and geometric standard deviations were converted to arithmetic means using equations in Higgins et al. (2008). Studies that provided both AM and GM were used to validate the accuracy of using such conversion (SI.2). Some studies only provided GM without geometric standard deviation or median values, and therefore these statistics could not be converted to arithmetic equivalents. While there were approximately 20% of studies that only reported statistics that could not be converted to arithmetic equivalents, these measurements were still included in the analysis as they were deemed to be informative. It is therefore important to interpret the statistical summaries with caution as it is likely that median or geometric summary statistics will have lower values than arithmetic means due to the log-normal nature of air pollution exposure (Avery et al., 2010; de Nazelle et al., 2017).

To provide an indication of how personal exposures related to ambient concentrations, a simple ratio of personal exposure divided by ambient concentration were summarised for studies that reported this metric. Studies that calculated the correlation (Pearson, Spearman or coefficient of determination) between personal exposure and ambient concentrations were recorded and discussed. The characteristics of groups monitored were also summarised by collating the time-activity patterns and proportion of PM_2.5_ exposures experienced during these activities.

## 3 Results

### 3.1 Study locations

**Fig. 1:**
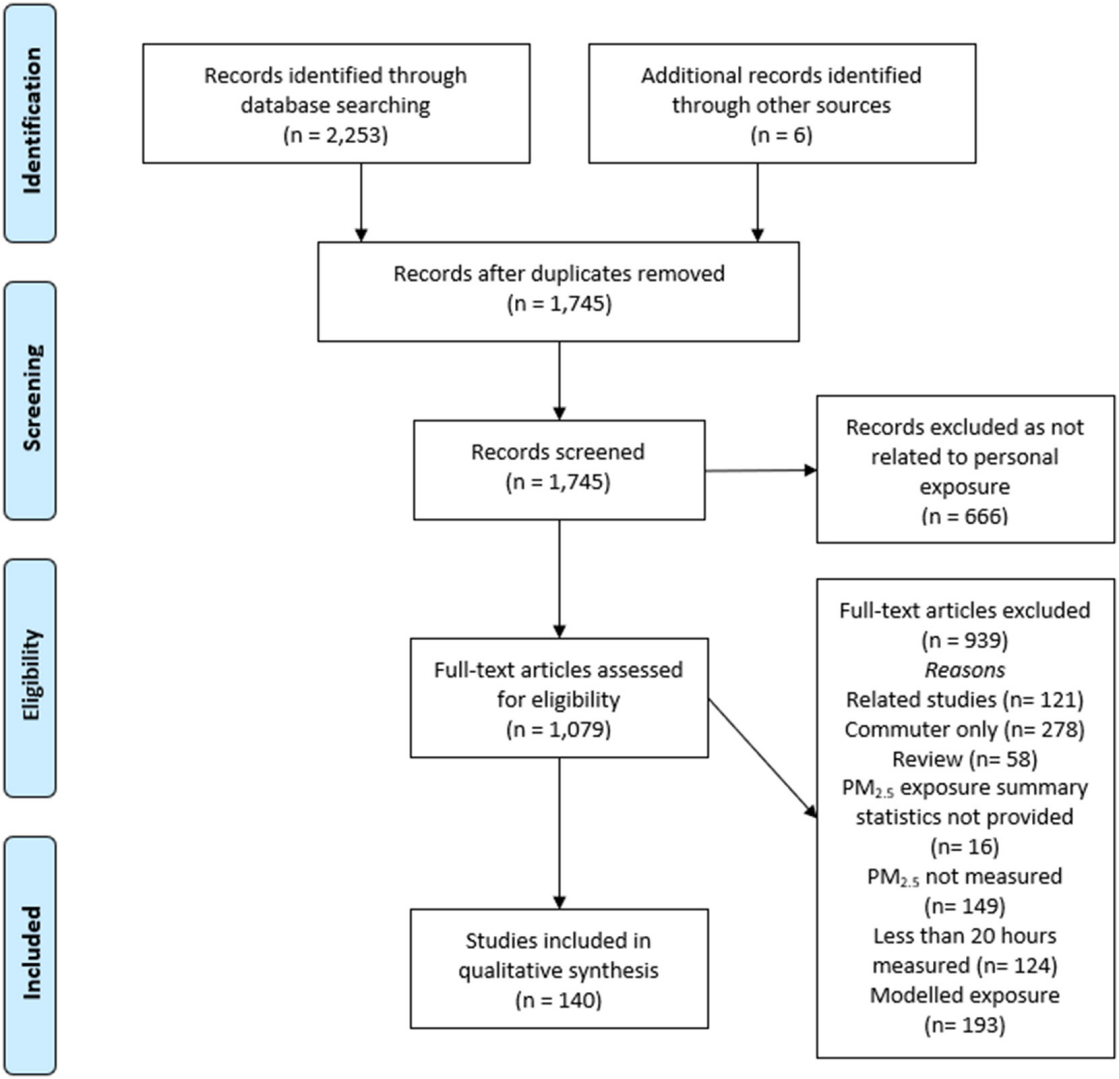
Flow diagram of study selection for personal PM_2.5_ exposure studies.

**Fig. 2:**
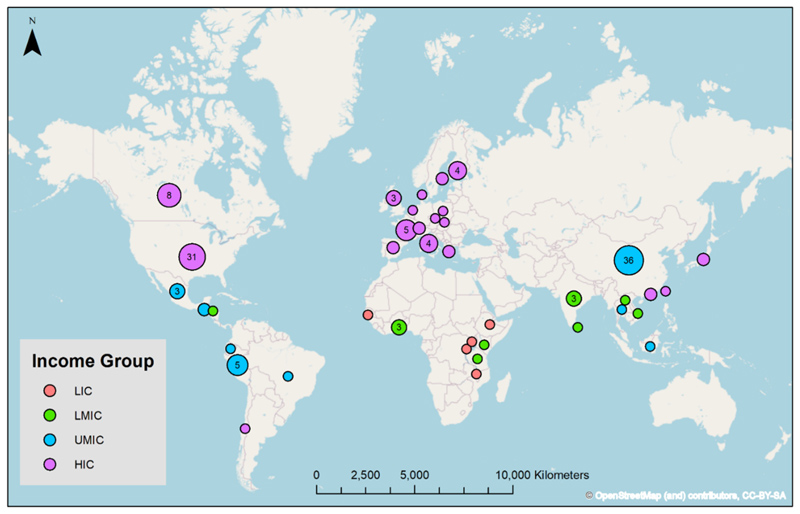
Geographical distribution of personal PM_2.5_ exposure studies summarised into low (LIC), lower-middle (LMIC), upper-middle (UMIC), and high (HIC) income countries (World Bank, 2020). The number of studies is indicated inside the dots except where two or fewer studies were conducted in each country.

From the 2259 titles that were identified, there were 140 studies that met our criteria (Fig. 1). Most studies collected personal exposure measurements to characterise various aspects of people’s exposure to PM_2.5_ (111 studies), however, some studies also collected measurements to better understand the health effects of exposure (29 studies). The studies were located across 40 countries in 128 unique cities or rural regions, with some studies conducting measurements in multiple locations (Table S1). In total, 74 studies were conducted in HICs, 50 in UMICs, 12 in LMICs, and 4 in LICs (Fig. 2). In terms of regions 49 studies were conducted in Asia, 45 in North America, 29 in Europe, 9 in Africa, and 8 in South America. The USA and China had the most studies conducted with more than 30 in each country. Canada (8 studies) was the third most common country studied. There was a noticeable absence of studies in North Africa, West Asia, and Oceania.

**Fig. 3:**
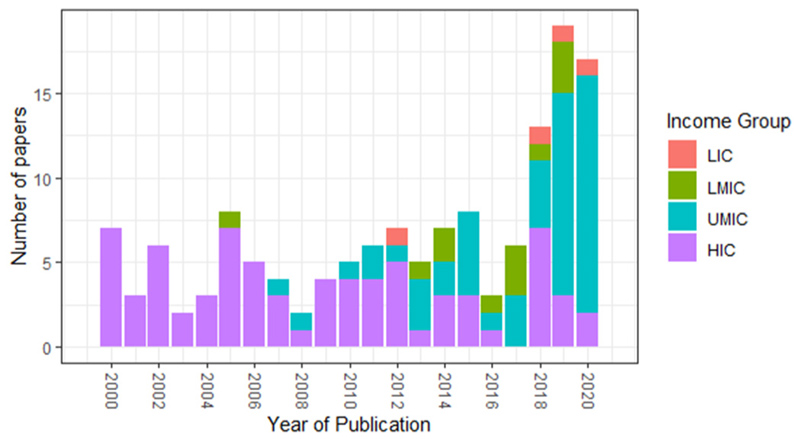
Number of papers published between 2000 and 2020, split between low (LIC), lower-middle (LMIC), upper-middle (UMIC), and high (HIC) income countries (World Bank, 2020).

There has been a rapid increase in studies published in the last 5 years with 58 studies published after 2015, compared to only 49 between 2000 and 2010 (Fig. 3). In the decade up to 2010, personal exposure studies were predominantly focussed in HICs. Since 2010, there has been an increasing number of studies in UMICs, predominantly in China, while the prevalence of HIC studies has decreased. There is still a sparse number of studies conducted in LMIC or LIC, with all but one study conducted after 2012. Due to the small number of studies, LMIC and LIC studies were analysed together for this review.

### 3.2 Population groups monitored

The total number of participants in each study ranged from our cut-off of 10 to 1330 individuals, with the median sample size being 56 participants. The majority of studies measured a single participant’s exposure for 24 or 48 h, however this varied between studies, with one study measuring exposure for 12 consecutive days (Koutrakis et al., 2005), while others undertook repeated measurements on the same participant across different seasons or before and after interventions (Table S1). Studies often summarised PM_2.5_ exposure into different group measurements based on location, season, gender, age, health status, occupation, and intervention type. In total 273 group measurements were summarised from the 140 studies. Over 63% of group measurements targeted exposures for adults, this included those in work, home workers, pregnant women, individuals with medical conditions, and university students (Table S3). Approximately 17% of groups measured focussed on children and infants with another 16% measured exposures of the elderly. The remaining studies did not have a defined target group or summarised exposures in a mix of two of the three age groups mentioned above (i.e. adult and child). Fifty-two of non HICs measurements (out of 136 group measurements) focussed on home workers, with the other main categories targeting the general working population (26%), primary or secondary school children (12%), elderly (11%), and university students (4%) (Table S4). For HIC measurements, over 37% focussed on the general adult population, with other popular categories monitored being vulnerable groups such as school children (18%), individuals with medical conditions (15%), elderly (12%), and pregnant women (5%).

Two-thirds of the summarised group measurements were conducted in urban locations, however, 89% of measurements in HICs were conducted in urban areas compared to only 52% in UMICs and 26% in LMIC and LICs (Table S5). This could be due to the higher proportion of the population living in rural areas in these lower income countries (United Nations, 2019) and also the likelihood that personal exposure studies focus on rural areas in lower income countries due to significant sources of indoor pollution in these areas through solid fuel cooking and heating (Quansah et al., 2017). Fourteen studies also investigated differences in personal exposure between interventions such as the use of indoor air filters or changing cookstoves to lower pollution appliances, again these were predominantly conducted in lower income countries (Barkjohn et al., 2020; Baumgartner et al., 2019; Brehmer et al., 2020; Chartier et al., 2017; Cynthia et al., 2008; Fitzgerald et al., 2012; Hartinger et al., 2013; Hill et al., 2019; Kirby et al., 2019; Lam et al., 2018; Liao et al., 2019; Maestas et al., 2019; McCracken et al., 2013; Pollard et al., 2018). Personal exposure measurements in a small proportion of studies (14%) were also compared between seasons, often comparing summer and winter or wet and dry seasons depending on location (Arhami et al., 2009; Baumgartner et al., 2011; Baumgartner et al., 2019; Brauer et al., 2000; Brown et al., 2009; Folino et al., 2009; Georgiadis et al., 2001; Hu et al., 2019; Koutrakis et al., 2005; Lachenmyer and Hidy, 2000; Li et al., 2019; Loo et al., 2020; Ohura et al., 2005; Pant et al., 2017; Sarnat et al., 2000; Sinaga et al., 2020; Sorensen et al., 2005; Van Ryswyk et al., 2014; Zhang et al., 2015).

### 3.3 Monitor types and methods

**Fig. 4:**
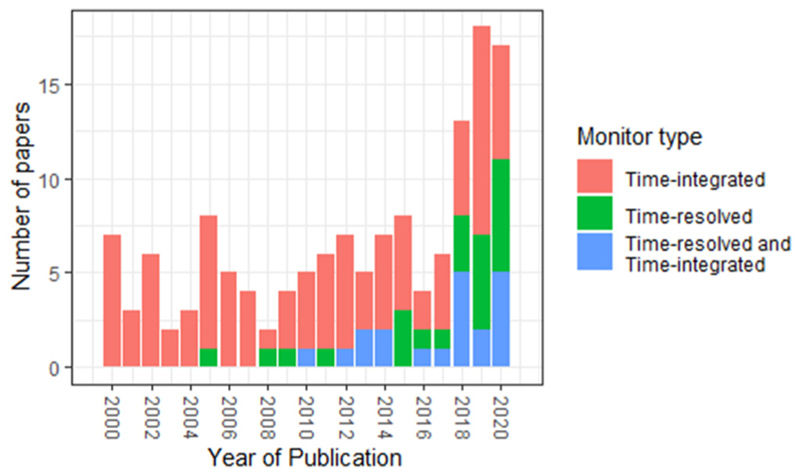
Number of papers published between 2000 and 2020, split between use of time-integrated, time-resolved or both types of monitors.

The monitors used to measure personal PM_2.5_ exposures were split between time-integrated monitors which collect samples on a filter over a specific time period (typically 24 or 48 h) and are gravimetrically weighed (Williams et al., 2000b) or time-resolved monitors, either using optical particle counters (OPC) or nephelometers (Hagan and Kroll, 2020). In total, 97 studies used time-integrated monitors, 23 used time-resolved while 20 used both types of monitors concurrently (Table S1). The resolution of exposure ranged from 10 s using time-resolved monitors to 96 h under time-integrated instrumentation. In the last five years, there has been an increasing number of studies that have employed time-resolved monitors (Fig. 4), with most studies including details on monitor accuracy and co-location procedures (Aunan et al., 2019; Barkjohn et al., 2020; Borgini et al., 2011; Buonanno et al., 2015; Chartier et al., 2017; Cynthia et al., 2008; Hu et al., 2019; Koehler et al., 2019; Lei et al., 2020; Liao et al., 2019; Lin et al., 2020; Pant et al., 2017; Sarnat et al., 2018; Sinaga et al., 2020; Sloan et al., 2016; Xia et al., 2019; Ye et al., 2020). For the 20 studies that used both types of monitors they did so to provide temporal and spatial detail of the monitoring period and also used the integrated monitor to check the accuracy of the time-resolved monitor (Arku et al., 2015; Delapena et al., 2018; Ducret-Stich et al., 2012; Gould et al., 2020; Lam et al., 2018; Lei et al., 2020; Li et al., 2019; Liao et al., 2019; Liu et al., 2020; Maestas et al., 2019; Okello et al., 2018; Pollard et al., 2018; Sarnat et al., 2018; Sloan et al., 2017; Smargiassi et al., 2014; Spira-Cohen et al., 2010; Van Ryswyk et al., 2014; Van Vliet et al., 2013; Xia et al., 2019; Ye et al., 2020). Eighty-eight studies recorded ambient fixed monitoring concentrations alongside personal exposure measurements, while 42 studies reported a correlation statistic between personal exposure and ambient concentrations (Table S1). Ambient concentrations were either measured by monitors outside people’s place of residence, a central monitor located in the participants’ community or a regulatory monitor which is part of a national or city monitoring network.

Regarding the quality of studies, just over a third of studies employed good QA/QC procedures and had sample sizes greater than 30 participants, with 48 studies rated high-quality, 73 studies rated moderate quality and only 18 studies rated low quality. As this was a subjective quality rating and a rigorous quality assessment has yet to be established for air quality exposure research, no studies were excluded based on their quality rating.

### 3.4 PM_2.5_ exposures reported

**Fig. 5:**
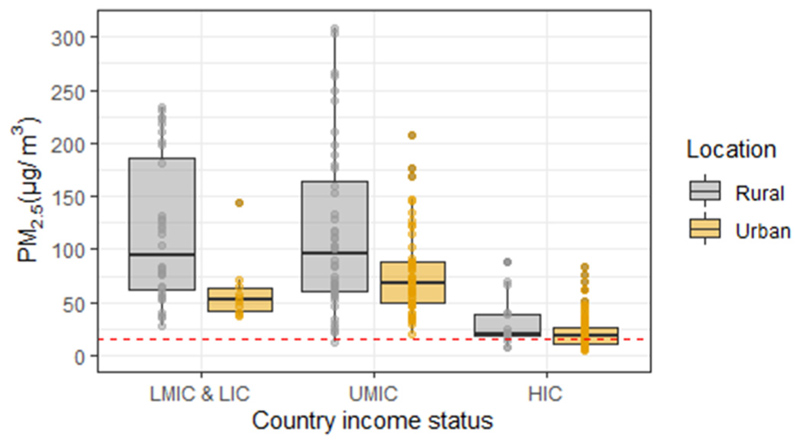
Summary of 273 personal PM_2.5_ group measurements separated by rural and urban locations across different countries income status. Bold horizontal black lines denote the median proportion; boxes extend from 25th to 75th percentile; vertical lines indicate 1.5 times the interquartile range. The dotted red line is the WHO 24-h guideline at 15 μg/m^3^. The y-axis excluded exposures >310 μg/m^3^, as a result, two measurements were not presented, an urban LMIC exposure of 484.0 μg/m^3^ in New Delhi, India (Pant et al., 2017) and a rural UMIC exposure of 451.0 μg/m^3^ in Taigu, China (Du et al., 2017).

For the 273 group measurements recorded, PM_2.5_ exposures ranged between 4.3 μg/m^3^ for elderly residents in Kuopio, rural Finland (Siponen etal., 2019) to 484.0 μg/m^3^ for non-smoking office workers during the winter in the megacity of New Delhi, India (Pant et al., 2017). The median exposure recorded was 37.2 μg/m^3^, with the 1st quartile 18.8 μg/m^3^ and the 3rd quartile 78.0 μg/m^3^, highlighting the variability in exposures measured between groups (Table 1). Median personal PM_2.5_ exposures were lowest in HICs (18.9 μg/m^3^) and highest in LMIC and LICs (78.0 μg/m^3^). PM_2.5_ exposure measurements in rural areas typically reported higher exposures than urban areas, particularly in lower income countries (Fig. 5, Table S5).

While WHO air quality guidelines are typically designated for ambient levels of pollution, they can also provide an indication of the magnitude of exposures experienced. There was only 1 group where average PM_2.5_ exposures were below the WHO ambient annual guideline of 5 μg/m^3^, although this increased to 48 groups compared to the WHO 24-h mean guideline of 15 μg/m^3^ (World Health Organisation, 2021b). To further highlight the inequality in exposure between countries, 34% of groups in HICs reported below the WHO daily mean guideline, compared to only 1% of UMICs and 0% of LMICs and LICs groups. (This was 71% of groups in HICs, 4% of UMICs and 0% of LMICs and LICs groups for the interim target 4/2005 24-h mean guideline of 25 μg/m^3^).

### 3.5 Exposure in high income countries

Personal PM_2.5_ exposures in HICs ranged between 4.3 and 88.0 μg/m^3^, with the median exposure at 18.9 μg/m^3^ (Table 1). The median participant size in HIC studies was 49 (interquartile range: 28 to 92 participants). The majority of studies in HICs were located in Europe and North America (84%), with one study from Chile and the remaining studies in Asia (Hong Kong, Taiwan, and Japan) (Table S1). The highest exposure recorded was in Banska Bystrica, a small town in Slovakia in 1997 for high school students and office and industrial workers (Brauer et al., 2000). However, the highest exposures that have been recorded in the last five years were exposures for females in Ceccano, rural Italy at 40.8 μg/m^3^ (Buonanno et al., 2015).

Half of the HIC studies (37 out of 74) reviewed reported details of time activity of participants, predominantly through the use of hand-written or electronic diaries (Adgate et al., 2003; Brauer et al., 2000; Buonanno et al., 2015; Chen et al., 2018; Ebelt et al., 2000; Ho et al., 2019; Jacquemin et al., 2007; Jedrychowski et al., 2006; Johannesson et al., 2007; Kim et al., 2006; Koutrakis et al., 2005; Lachenmyer and Hidy, 2000; Lai et al., 2004; Miller et al., 2019; Minguillon et al., 2012; Mohammadyan, 2011; Montagne et al., 2014; Mosqueron et al., 2002; Noullett et al., 2010; Ohura et al., 2005; Rodes et al., 2010; Sarnat et al., 2005; Siponen et al., 2019; Sørensen et al., 2005; Spira-Cohen et al., 2010; Suh and Zanobetti, 2010; Van Ryswyk et al., 2014; Williams et al., 2000a; Williams et al., 2003; Wu et al., 2005, 2006), although more recently GPS location (Cunha-Lopes et al., 2019; Koehler et al., 2019; Sloan et al., 2016, 2017; Steinle et al., 2015; Zamora et al., 2018) has also been used to determine participant activity (Table S2). Time spent indoors during a day ranged from 81% to 98%, with median time spent indoors being 90% (Table S2). Activities in studies were typically split between time spent at home (median 69%), at work or school (21%), commuting (5%) and other. Despite recording activity patterns, few studies identified the proportion of PM_2.5_ exposure during each activity due to the use of time-integrated monitors, with only three studies recording the proportion of exposure in these environments using high-resolution monitors (Buonanno et al., 2015; Van Ryswyk et al., 2014; Wu et al., 2005). The proportion results of these studies are conflicting, however generally time spent at home resulted in a lower proportion of daily exposure, while commuting had opposite results. Time spent and the proportion of PM_2.5_ exposures at school/work were similar.

The predominant sources of PM_2.5_ exposure reported in HICs were second hand smoke (SHS), particularly in the early 2000s (Ebelt et al., 2000; Gauvin et al., 2002; Georgiadis et al., 2001; Lai et al., 2004; Mosqueron et al., 2002; Oglesby et al., 2000; Sørensen et al., 2005) and ambient pollution infiltrating indoors (Arhami et al., 2009; Borgini et al., 2011; Ebelt et al., 2000; Janssen et al., 2000; Rojas-Bracho et al., 2002; Sarnat et al., 2000; Van Roosbroeck et al., 2007; Williams et al., 2003). While a number of studies identified SHS or active smoking as a source of pollution, 80% of HICs studies measured non-smoking participants (Table S1). SHS as a source is thought to have largely decreased in HICs in recent years due to second-hand smoking campaigns highlighting the risk (Jain, 2016). Studies also suggested that traffic emissions infiltrating indoors was an important source of PM_2.5_ exposure for those living near busy roads (Brown et al., 2009; Koutrakis et al., 2005; Spira-Cohen et al., 2010; Zamora et al., 2018). However, one recent study did not find a difference in personal exposure between university students living closer or further away from highways (Sarnat et al., 2018), with results suggesting the similarity between exposures was due to improving vehicle emission standards in America. There were also several studies that highlighted sources of PM_2.5_ exposure from cleaning and cooking (Adgate et al., 2003; Buonanno et al., 2015; Jacquemin et al., 2007; Meng et al., 2005; Steinle et al., 2015; Van Ryswyk et al., 2014; Xia et al., 2019; Yang et al., 2019; Zamora et al., 2018). Some studies suggested that commuting and time spent in restaurants had a significant influence on exposure despite the relatively short time periods that participants spent in these environments (Chen et al., 2018; Ducret-Stich et al., 2012; Mohammadyan, 2011; Sloan et al., 2016). Indoor wood and candle burning were also discussed as sources but were thought to have a minimal effect on personal exposure for people in HICs (Molnar et al., 2005; Siponen et al., 2019). The majority of PM_2.5_ sources for personal exposure that have been identified were from observations as opposed to robust quantitative or qualitative analysis. Therefore, due to the array of differences in methodologies, time periods and locations, there was conflicting evidence to suggest which sources were the most influential for personal exposure. However, the variability in exposure could also be the result of the diversity of groups measured and locations which were monitored.

Similar exposures were measured in children and adults in HICs (medians = 19.1 μg/m^3^ and 21.0 μg/m^3^ respectively), with elderly participants reporting slightly lower exposures (14.3 μg/m^3^), hypothesised due to increased time spent indoors (Table S7). There were only nine measurements that reported female only exposures and one measuring males, therefore, comparisons of exposures between gender could not be made (Table S8). While the majority of studies in HICs were conducted in urban areas, measurements of personal exposures in rural (median = 20.8 μg/m^3^) and urban areas (median = 18.6 μg/m^3^) reported similar values (Table S5).

The ratio between personal exposures to ambient concentrations was often higher than 1 (median = 1.2), with 76 out of 111 groups recording higher personal exposures compared to ambient measurements and ratios ranging between 0.4 and 4.0 (Table S6). Unsurprisingly the highest personal to ambient ratios were often related to studies that measured the absolute highest personal exposures (Brauer et al., 2000; Lioy et al., 2011), suggesting a larger source of indoor generated PM_2.5_ in these studies. Despite ambient pollution levels slightly decreasing in the last two decades in HICs (Cohen et al., 2017), no obvious trend in personal to ambient ratio was observed over time. In terms of how well ambient concentrations were correlated to personal exposures, results were mixed. Studies measured this relationship by calculating a Pearson’s or Spearman’s correlation coefficient or R^2^ (42 HICs groups reported these results) and ranged from 0.01 to 0.84 (Table S1).

There did not appear to be much difference in exposures between seasons in HICs with similar median exposures reported for summer (20.8 μg/m^3^), winter (18.5 μg/m^3^) and autumn (20.1 μg/m^3^) (Table S9). However, it is likely that the difference in exposures between seasons will be affected by the environment at each study location and therefore these pooled results across locations should be interpreted with caution.

### 3.6 Exposure in upper-middle income countries

Personal exposure in UMICs ranged between 13.0 and 451.0 μg/m^3^, with the median exposure measured at 74.3 μg/m^3^, almost four times higher than HICs (Table 1). The median participant size in UMIC studies was 67 (interquartile range 40 to 114 participants). Seventy-five percent of these studies were conducted in Asia (36 in China, one in Indonesia, and one in Thailand), with the remaining studies in South America (*n* = 7) and North America (n = 5) (Table S1). The lowest exposures were measured in rural Ecuador on home workers who cooked using LPG during the wet season in 2019 (Gould et al., 2020), and was the only UMIC measurement below the WHO daily guideline of 15 μg/m^3^. The highest exposures were measured for residents who used biomass for heating and cooking in Taigu, rural China in the winter (W. Du et al., 2017). Though, these exposures were over 100 μg/m^3^ higher than the next two highest studies, one in rural Guatemala which measured mean PM_2.5_ exposure of 266.0 μg/m^3^ for female homeworkers (McCracken et al., 2013) and another study in Sichuan Province, rural China which measured mean exposures of 308.2 μg/m^3^ in the winter (Baumgartner et al., 2019).

Only 13 out of 50 studies reported the time-activity of participants in UMICs (Chen et al., 2020; Cortez-Lugo et al., 2015; Du et al., 2010; Hu et al., 2019; Lei et al., 2020; Li et al., 2019; Liao et al., 2019; Lin et al., 2020; Sinaga et al., 2020; Tovalin-Ahumada et al., 2007; Xu et al., 2018; Ye et al., 2020; Zeng et al., 2020), the results were similar to HICs with time spent indoors ranging from 67% to 97%, with a median of 87% (Table S2). However, it is important to note that the majority of studies that recorded participants time-activity were in urban centres (Chen et al., 2020; Cortez-Lugo et al., 2015; Du et al., 2010; Lei et al., 2020; Li et al., 2019; Lin et al., 2020; Sinaga et al., 2020; Zeng et al., 2020), so may not reflect activity trends in rural settings. One study compared the time-activity between elderly urban and rural residents and found that the rural residents spent more time indoors compared to urban (Hu et al., 2019), however further studies are needed to confirm whether this is observed in all areas or specific to this study group. Time spent in different microenvironments was again similar to HICs with a median of 69% time spent at home, 1% of time spent commuting and 31% of time spent at work/school. Only one study reported the proportion of PM_2.5_ exposure in different microenvironments and found a similar proportion of exposure and time spent in both indoor and outdoor environments (Hu et al., 2019).

The primary sources of PM_2.5_ hypothesised in UMIC studies were from cooking and heating activities, where biomass such as wood or coal were often used (Aunan et al., 2019; Baumgartner et al., 2011, 2019; Cynthia et al., 2008; Du et al., 2010; Huang et al., 2017; Liu et al., 2018; Orakij et al., 2017; Secrest et al., 2016; Shan et al., 2014; Wu et al., 2020; Xu et al., 2018; Ye et al., 2020). One study in Shanghai, China suggested that 10–15% of their participants daily PM_2.5_ exposure was the result of cooking (Zeng et al., 2020). A number of studies compared personal exposure between groups using alternative stove types such as stoves with and without chimneys (Fitzgerald et al., 2012; Hartinger et al., 2013) and biomass versus LPG or electric stoves (Du et al., 2017; Gould et al., 2020; Hu et al., 2019; Liao et al., 2019; Pollard et al., 2018; St Helen et al., 2015), with results suggesting that PM_2.5_ exposures were lower when cookstoves with chimneys or cleaner fuels were used. Some studies mentioned traffic, ambient biomass emissions, and dust as sources of personal PM_2.5_ exposure (Liang et al., 2019; Shan et al., 2014; Xu et al., 2018; Zhang et al., 2015), while SHS or active smoking as a source was discussed in only a few studies (Huang et al., 2017; Secrest et al., 2016). One study mentioned mosquito coils as being a significant source of personal exposure (Sinaga et al., 2020). Unlike HICs, ambient pollution infiltrating indoors was not discussed as a significant source, apart from recent studies in urban or periurban areas in China (Barkjohn et al., 2020; Brehmer et al., 2020; Chen et al., 2020; Fan et al., 2020; Lei et al., 2020; Li et al., 2019; Lin et al., 2020; Shang et al., 2020). There were also studies that investigated exposures for different occupational groups with park workers having lower exposures compared to street workers in Sao Paulo (Cozza et al., 2015), office workers having a third lower exposures compared to street vendors and taxi drivers in Mexico City (Tovalin-Ahumada et al., 2007) and office workers having slightly lower exposures than taxi drivers in Beijing (Du et al., 2011), with all results suggesting that working nearer to traffic emissions resulted in higher personal PM_2.5_ exposures.

For these UMICs studies, there was a greater focus on gender differences in exposure due to the identification of household sources of pollution from cooking related activities in the kitchen. There were 41 measurements that recorded female exposures and they found median exposures of 90.0 μg/ m^3^. However, there were insufficient studies that investigated male only exposures (six measurements) to make fair comparisons between genders (Table S8). The higher female exposures compared to all UMIC measurements suggests that females are potentially disproportionately affected by PM_2.5_ exposure. While it is difficult to directly compare these exposures due to differences in methodologies between studies, one study found higher exposures in males compared to females, which was hypothesised due to the prevalence of male smokers (Huang et al., 2017). Children and the elderly had lower median exposures (65.0 μg/m^3^) compared to adults (84.2 μg/m^3^), with the higher exposures possibly being explained due to the propensity of studies that focussed on adult home-workers who are exposed to elevated emissions while cooking (Table S7). However as there were only 19 measurements on children and elderly, compared to 72 for adults, further studies are needed to confirm this observation.

There appeared to be a larger rural-urban divide in exposures for UMICs compared to HICs, with median urban measurements at 67.6 μg/m^3^ compared to rural studies at 100.5 μg/m^3^ (Table S5). This difference is further exhibited with studies that compared personal exposures to ambient concentrations. The urban UMIC ratio (median = 1.1) was similar to HICs, however, in rural areas this ratio was much higher (median = 5.1) (Table S6). The high rural ratio was substantially affected by Baumgartner et al. (2019) study which reported eight personal to ambient ratio measurements between 3.9 and 14.6 (Table S1), however even after removing this study from the analysis the rural personal to ambient ratio was 1.8, still higher than the urban UMIC ratio. A potential explanation for this trend is that for countries that have recently experienced rapid development such as China, the rural population is more highly exposed to PM_2.5_ due to reliance on traditional biomass heating and cooking fuels compared to modern urban centres where electrical or gas appliances are available (Lee et al., 2021). However, it is also important to note that both the highest and lowest exposures for UMICs were in rural areas, highlighting minimal indoor sources of PM_2.5_ in some rural locations. Only nine groups reported correlations between personal and ambient exposures, again the range was similar to HICs (0.03 to 0.81) with no discernible trend behind strong or weak correlations (Table S1).

Compared to HICs there appeared to be a larger difference in seasonal exposures in UMICs with higher median exposures in winter (128.7 μg/m^3^) comparedwith summer (66.8 μg/m^3^) (Table S9), this difference is likely due to the increased biomass or coal burning for heating during the winter (Baumgartner et al., 2019). While there was also a difference in exposures identified between dry (104.0 μg/m^3^) and wet seasons (26.2 μg/m^3^) (Table S9), the only study which was conducted over the wet season was in Ecuador (Gould et al., 2020), which also was the study where participants had the lowest exposure among UMICs.

### 3.7 Exposure in lower-middle income and low income countries

Due to the small number of studies, LMICs (12 studies) and LICs (4 studies) were grouped together to analyse exposures in these countries. Nine of these studies were conducted in Africa and six studies in Asia (three being in India) (Table S1). Only one study in Honduras was outside of these continents. Although only 16 studies were conducted, 43 different groups were measured within these studies. The median participant size in these studies was 60 (interquartile range 47 to 220 participants). Personal PM_2.5_ exposure in these countries had a wide range from 28.4 to 484.0 μg/m^3^, with the median measurement being 78.0 μg/m^3^ (Table 1). The lowest exposures were measured in the rural area Kikati, Uganda, for male school children (Okello et al., 2018). The next lowest exposures were measured in an intervention study in Kenya which found personal exposure at 35.0 μg/m^3^ for high school children who were provided with solar lamps compared to exposure of 132.0 μg/m^3^ when they used kerosene lamps (Lam et al., 2018). The highest exposures were measured in a pilot study for office workers in New Delhi, India in winter (Pant et al., 2017), although exposures in the summer were significantly lower at 53.9 μg/m^3^. Therefore, the significant source of this exposure was largely thought to be due to ambient pollution levels rather than any indoor sources as ambient PM_2.5_ concentrations were reported at 545.0 μg/m^3^ in winter. The highest exposures measured in New Delhi were more than double the exposure of the next highest measurement; females who used cow dung to cook in Kumbursa, Ethiopia at 234.0 μg/m^3^ (Okello et al., 2018), although this study was conducted in the rainy season.

Only three studies recorded the participants time-activity in these countries. A study in India found similar findings to HICs with approximately 90% of time spent indoors in Mysore (Andresen et al., 2005), while 80% of time was spent at home for participants in Ho Chi Minh City (Mehta et al., 2014). Conversely, participants in Laos only spent 75% of their time indoors (Hill et al., 2019).

The identified sources of pollution were similar to the rural UMIC studies, with the focus almost solely on indoor sources of pollution, particularly while cooking (Andresen et al., 2005; Chartier et al., 2017; Delapena et al., 2018; Dionisio et al., 2012; Hill et al., 2019; Kirby et al., 2019; Lam et al., 2018; Van Vliet et al., 2013). Again, there were a handful of intervention studies that measured the effect on personal PM_2.5_ exposures of new cookstoves such as kerosene and gas (Andresen et al., 2005), different designs of biomass stoves (Chartier et al., 2017; Kirby et al., 2019) and electric stoves (Hill et al., 2019). Another study also investigated personal exposures using different lamp types (Lam et al., 2018). Like the UMIC studies, in most cases, the use of modern appliances was found to provide some reduction in personal exposure, although one study did not find an improvement when using new stoves (Kirby et al., 2019). In urban areas, traffic sources were also discussed, however, they were still not thought to be a significant contributor to personal exposure in these countries compared to cooking sources (Delapena et al., 2018; Mehta et al., 2014; Pant et al., 2017). Three studies focussed on the differences between exposures between genders (Arku et al., 2015; Okello et al., 2018; Sanchez et al., 2020) with one in Ethiopia and Uganda finding over four times higher exposure in females compared to males, again, likely related to time spent in the kitchen (Okello et al., 2018). Arku et al. (2015) also found the same trend in Accra, Ghana with female school children having higher exposures compared to their male counterparts. The other study in rural Telangana, India found no such difference in exposures between genders with both reporting exposures around 60 μg/m^3^, hypothesised due to females higher exposure experienced using biomass in the kitchen offset by males smoking and working in construction or industry (Sanchez et al., 2020).

Similar to the UMIC studies, there were higher personal exposures measured in rural areas (median 93.9 μg/m^3^) compared to urban (median 53.9 μg/m^3^), with exposures measured largely similar to UMICs (Table S5). However, as only 11 measurements were conducted in urban centres across four countries (Vietnam, India, Ghana and Tanzania) in LMICs and LICs, more studies are needed to be able to make robust comparisons. The median personal to ambient ratio was 1.5, however as only 13 groups presented ambient measurements (Arku et al., 2015; Delapena et al., 2018; Hill et al., 2019; Mehta et al., 2014; Pant et al., 2017; Sanchez et al., 2020; Van Vliet et al., 2013), there is uncertainty on how reflective these ratios are for LMICs and LICs in general (Table S6). There were only three studies that assessed the correlation between personal exposure and ambient concentrations, with correlations ranging from 0.4 to 0.7 (Arku et al., 2015; Mehta et al., 2014; Pant et al., 2017) in Accra, Ho Chi Minh City and New Delhi.

Median exposures were higher for levels recorded in dry seasons (104.0 μg/m^3^) compared with wet seasons (82.1 μg/m^3^) in LMICs and LICs (Table S9). The seasonal difference in exposures is assumed to be due to rainfall removing particles from the air during the wet season and dust being more prevalent during the dry season (Arku et al., 2015).

## 4 Discussion

Overall, personal PM_2.5_ exposures were lower in HICs compared with other countries, with UMICs exposures being slightly lower than those measured in LMICs or LICs. There was similar exposures recorded between rural and urban participants in HICs, but there were noticeably higher exposures recorded in participants in rural areas compared to urban areas in non-HICs, likely due to predominant indoor sources of PM_2.5_ in rural locations. There was insufficient evidence found on PM_2.5_ exposures differing between genders, as there was a distinct absence of studies that reported male exposures to be able to make fair comparisons. In terms of differences in exposures with age, studies found lower exposures experienced by the elderly compared to adults, which was potentially due to the longer period the elderly spends indoors. Our review also highlighted that there is still a lack of studies conducted in lower income countries with less than 12% of the studies reviewed in LMIC and LICs, but trends show an increasing number of studies in these countries in the last five years.

This review also highlighted that personal exposure measurements are an important tool to characterise people’s exposure, identify determinants of exposure and can assist with understanding the health effects of shortterm variations in air pollution. It is, however, important to recognise that due to the practicalities with collecting a personal exposure dataset which is representative of the population, personal monitoring cannot replace epidemiological studies which use estimates of exposure for millions of people over multiple years. This is reflected in the review as the study with the largest sample size had just over 1300 participants (Rylance et al., 2020). Furthermore, personal exposure studies reviewed typically undertook measurements for short time periods (24–48 h) and therefore there is uncertainty whether these measurements would be reflective of personal exposures over the longer time periods which are most relevant to the health impacts of chronic air pollution.

Time-activity records in the reviewed studies found that participants in HICs spent approximately 90% of their time indoors, with 70% spent in their residence, these trends did not appear to be different compared to urban UMICs. However, there were still very few studies that recorded the daily activity patterns of exposure in populations in non-HICs. The activity patterns of participants in rural areas were also not widely reported. A better understanding of activity patterns and the proportion of exposures in these areas is important to identify activities where exposures are the highest.

There was a wide range of sources identified for personal PM_2.5_ exposure. In HICs, SHS, ambient pollution infiltrating indoors, and traffic emissions were found to be the dominant contributors to daily exposure. While, in non-HICs, cooking and heating with biomass and coal were noted as the most important sources. Traffic emissions, road dust, and kerosene lamps were also other noted sources. However, it is important to note that the identification of sources in the reviewed studies was largely observational or tested when comparing different groups (i.e. changing from biomass to LPG stoves or comparing smokers and nonsmokers). Only a small proportion of studies quantified the contribution of these sources to daily PM_2.5_ exposure measuring the chemical elements of PM_2.5_ and utilising positive matrix factorisation (Larson et al., 2004; Shang et al., 2020; Zhang et al., 2015) or employing linear regression methods (Brown et al., 2009; Chen et al., 2018; Cortez-Lugo et al., 2015; Gauvin et al., 2002; Jedrychowski et al., 2006; Mehta et al., 2014; Mohammadyan, 2011; Mosqueron et al., 2002; Sanchez et al., 2020; Sinaga et al., 2020; Van Ryswyk et al., 2014). The variety of PM_2.5_ sources emphasises that a one size fits all approach to reduce exposure in countries is unlikely to be effective, highlighting the importance of conducting local personal exposure studies.

Interventions such as the use of filters or different stove types were also widely tested in non-HICs to assess their effectiveness in reducing personal exposure to PM_2.5_. Stove interventions in lower income countries have generally found that they yield small air pollution exposure reduction benefits, however, are still unlikely to result in exposures meeting WHO guidelines (Baumgartner et al., 2019; Quansah et al., 2017; Thomas et al., 2015). The small exposure reduction observed is often due to interventions using the same existing cooking fuels only with increased ventilation (such as adding a chimney to the cookstove). Using cleaner fuels such as gas or electricity is thought to be more beneficial for reducing PM_2.5_ exposures from cooking (Quansah et al., 2017). Furthermore, participant’s short-lasting commitment to newly adopted behaviours has also been reported as a common problem in studies, limiting the impact of the interventions, with participants reverting back to traditional cooking means after the research has been completed (Baumgartner et al., 2019; Pillarisetti et al., 2014; Quansah et al., 2017). The adoption of initiatives that actively involve participants in the research process such as gathering their own personal exposure data has the potential to raise air pollution awareness and hence people’s understanding of the problem (Commodore et al., 2017; European Environment Agency, 2020; Varaden et al., 2021). This, in turn, can lead to sustained changes in behaviour to reduce exposure to harmful pollutants for people in these countries.

In general, personal exposure was found to be slightly higher than ambient fixed monitoring concentrations across all country’s levels of income. This suggests that there was an influence of indoor and commuting sources of pollution on PM_2.5_ exposure in the majority of studies (Fan et al., 2017). Other reviews found the median difference between personal exposures and ambient monitors in HICs was zero, with about 50% of personal exposures higher than ambient concentrations (Avery et al., 2010). What is interesting to note in this review was the large difference between urban (median 1.1) and rural (median 5.1) personal to ambient ratios in UMICs. This is likely due to the recent modernisation of heating and cooking infrastructure in urban centres in these countries, particularly in China (Lee et al., 2021). Furthermore, the majority of studies conducted in rural areas are specifically targeted to locations where household air pollution is high, resulting in higher personal exposure compared to ambient concentrations.

This review further suggests that fixed monitors may not be a good proxy for short term personal exposure, as there was inconsistency between studies in whether fixed monitors are good or bad predictors. There did not appear to be any uniform explanation as to why some ambient concentrations were strong or weak predictors of personal exposure, but the variation could be due to participants’ activity patterns and occupation, variation in ambient pollution infiltrating indoors, the season monitored, location of ambient monitor and the local environment monitored. In the early 2000s, some studies justified the use of ambient monitors as proxies for personal exposure in HICs due to high temporal correlations reported (Janssen et al., 2000; Sarnat et al., 2001; Williams et al., 2000b). However, recent reviews have highlighted that using ambient concentrations to estimate populations exposures from outdoor sources introduces measurement error (Evangelopoulos et al., 2020), suggesting that personal exposure studies are important to undertake for accurate exposure assessment. It is also important to note that the comparisons between ambient concentrations and personal exposure measurements are only for short time periods. Therefore, while fixed monitors may not be a good proxy for short term personal exposure measurements, there is uncertainty whether this result would also be true for personal exposures over a longer time period (i.e. annual exposure). While indoor environments are an important contributor to an individual’s exposure, sampling only indoor air has also been observed as a poor predictor of personal exposure, further highlighting the importance of personal exposure studies for accurate exposure assessment (Dias and Tchepel, 2018).

## 5 Limitations

The current review has some limitations. The primary focus of the studies reviewed varied widely, resulting in different methodologies, groups and durations of personal exposures measured. Therefore, a direct comparison of PM_2.5_ exposures between studies should be interpreted with caution. Furthermore, the specific aims of each study may skew an accurate representation of a group’s exposure, for example, successful interventions, such as alternative stove use would have reduced participants exposures. Instead, the exposures summarised in this review should act as a guide and provide insight into the current research gaps for personal PM_2.5_ exposure studies.

We only searched two electronic databases and included studies that were published in English. However, we believe we have covered the majority of personal PM_2.5_ exposure studies by also checking references in the studies reviewed to cover any other studies which may have been missed. Due to insufficient access to language resources, cost and time we did not include non-English studies in this review. While some Chinese journals have English-translated abstracts, the information provided in the abstract alone was deemed insufficient for obtaining the details needed for this review. However, we acknowledge the importance of non-English studies, and they should be considered for inclusion in future reviews. There were inconsistencies between studies reporting the calibration or quality control procedures for personal monitors used. Therefore, while comparability between participants exposures within a study may be viable, absolute measurements of PM_2.5_ exposure may be inaccurate.

While we assigned a quality assessment to each paper, this was based on subjective criteria, and no studies were rejected on this basis. It is likely that the results from those studies that were assigned a ‘low’ quality standard had high levels of uncertainty, which may have led to biased conclusions. A standardised framework to assess the quality of personal exposure studies would be useful to improve the certainty of evidence synthesised.

We employed a relatively arbitrary way to summarise exposures by splitting countries into different income bands. Countries within the income bands are diverse and have different personal characteristics which cannot be easily generalised. However, the analysis was conducted in this way to highlight the relative inequality of PM_2.5_ exposure and monitoring.

For comparability, our review was restricted to personal exposure studies which reported greater than 20 h of monitoring. We note that there are studies that have conducted personal exposure measurements over a shorter length of time which may include important exposure characteristics of populations that have not been reported in this review.

## 6 Recommendations for future research

In general, more personal exposure studies need to be conducted to understand where and when individuals are exposed to the highest levels of pollution and what types of interventions would be effective in reducing PM_2.5_ exposure to a wider range of the population. Further studies which allow for a better estimation of sources of PM_2.5_ exposure such as the use of chemical composition or X-ray fluorescence on samples would provide more robust evidence on the sources of pollution and therefore more effective interventions could be formulated to reduce exposure.

The review highlighted that further personal PM_2.5_ studies in LICs and LMICs are needed, as the variability, characteristics, and time-activity of exposure in these countries are still largely unknown. The literature available in LMICs and LICs is insufficient, and there is a need for more research to be conducted to reduce the inequality experienced by these countries in relation to air quality. It is also important for studies in these areas to characterise people’s exposures who work outside their home as the majority of studies to date have focussed on home workers. A better knowledge of exposures experienced in these areas would allow for better risk and health impact assessments of air quality in these countries. Furthermore, there were few studies which measured exposures to the most vulnerable age group, infants (Royal College of Physicians of London, 2016), studies on this group should be included in future personal exposure research. There was also a noticeable absence of personal PM_2.5_ exposure studies in North Africa and the Middle East despite high ambient levels of pollution (Health Effects Institute, 2020), and therefore personal exposure studies in these locations would also be an important contribution to the literature.

Increasing the use of optical high-resolution sensors for PM_2.5_ exposure studies should be encouraged. Not only does this provide further data on the characterisation of exposure, but there is also less infrastructure needed compared to time-integrated gravimetric devices which require specialised laboratories, sensitivity scales, and clean weighing rooms which may not be available in some countries (Okello et al., 2018). However, it is important that researchers conduct rigorous accuracy testing of these monitors before deployment, ideally with comparison to a reference monitor in a similar environment to the research setting. In the absence of a reference monitor, an inter-comparison precision test of devices may also be sufficient (i.e. colocating all the sensors next to each other in a polluted environment). Ideally, longer study periods of exposure are needed instead of one off 24–48 h samples, due to the variability of exposures reported within and between participants. There is still uncertainty surrounding the optimal number of repeated measurements needed to robustly characterise a populations exposure however consideration should be made to incorporate measurements across different seasons and time-activity patterns. Other studies have suggested that within subject exposure variability was three times greater than between subjects in LMICs (Clark et al., 2013; Dionisio et al., 2012; Lee et al., 2021). This indicates that repeated exposures of participants over time are needed to obtain a more accurate understanding of an individual’s typical exposure. Undertaking these repeated exposure measurements will also be important to understand the differences in exposure and ambient concentrations over longer time periods.

For HICs, while several studies highlighted that cooking, cleaning and heating were likely to contribute to indoor sources of PM_2.5_ exposure, there have been few studies that were able to quantify how much these sources contribute to daily exposure and if interventions which focus on reducing the indoor sources of exposure would be beneficial. The use of time-resolved monitors could assist in the quantification of indoor sources of pollution in these settings. While females have been suggested to be disproportionately affected by PM_2.5_ exposure, particularly in lower income countries (Okello et al., 2018), there have been minimal studies which have directly compared exposures between genders across all countries. Therefore, the reporting of exposures separated by gender is also encouraged (Schiebinger et al., 2016) and further studies are still needed to provide conclusive evidence of exposure differences between genders.

The review highlighted that UMICs have the greatest exposure difference between rural and urban areas. Further studies which investigate the differences in exposure in the same location (i.e. comparing urban versus rural locations in the same province or state), may provide insight into policies that can effectively reduce exposures in these rural areas. This knowledge could possibly be applied for effective ways to reduce exposures in LMICs and LICs. Further robust personal exposure intervention studies involving communities should be undertaken to be able to quantify the effectiveness of exposure reduction strategies and prioritise ones that have the biggest health and cost-benefit.

## 7 Conclusion

There has been a growing literature of personal PM_2.5_ exposure studies, revealing wide variance in exposures recorded even within the same country, and highlighting severe inequalities in geographical and social population subgroups. While ambient monitors are important for regulatory means, further personal exposure studies are needed for accurate exposure, risk and health impact assessment. There is an acknowledgement of the inequality faced by lower income countries with respect to the health effects of air pollution, however, this is also true of the frequency of personal exposure research undertaken in these locations. To provide a more equitable future, more personal exposure studies are needed which can provide a better understanding of the characteristics, magnitude, and awareness of pollution exposures specific to these countries. Studies that can identify practical and economically viable interventions that populations are willing to engage in to reduce exposures will also assist in avoiding the traditional pathway of rapid economic growth leading to air quality degradation that has previously afflicted HICs.

## Supplementary Material

Supplementary information

Supplementary tables

## Figures and Tables

**Table 1:** Summary statistics of personal PM_2.5_ exposure measurements split across country income status.

Income status	Number of studies	Number of groups measured	Personal PM_2.5_ exposure (μg/m^3^)				
			Minimum	1st quartile	Median	3rd quartile	Maximum
HIC	74	137	4.3	11.8	18.9	26.7	88.0
UMIC	50	93	13.0	53.0	74.3	122.8	451.0
LMIC & LIC	16	43	28.4	54.0	78.0	138.0	484.0
**Total**	**140**	**273**	**4.3**	**18.8**	**37.2**	**78.0**	**484.0**

HIC: High income country, UMIC: Upper-middle income country, LMIC: Lower-middle income country, LIC: Low income country as defined by World Bank (2020).

## Highlight

- Studies which conducted personal PM_2.5_ exposure monitoring were reviewed.
- Personal PM_2.5_ exposure studies have exponentially increased since 2015.
- Inequalities in magnitude of exposure were identified by country income status.
- Different sources of PM_2.5_ exposure by country income status were summarised.
- Recommendations to advance personal exposure research are discussed.
