# Supplementary material for: Comparing human exposure to fine particulate matter in low and high-income countries: A systematic review of studies measuring personal PM2.5 exposure
Source: Sci Total Environ. Author manuscript; Available in PMC 2023 Sep 15. (PMC7615091; doi:10.1016/j.scitotenv.2022.155207)
Supplement: Supplementary information [file EMS187688-supplement-Supplementary_information.docx]

**Comparing human exposure to fine particulate matter in low and high-income countries: A systematic review of studies measuring personal PM_2.5_ exposure**

**Supplementary Information**

1. **Systematic Review Protocol**

**Review Question**

What are the differences in personal PM_2.5_ exposures experienced between populations of low and high-income countries?

**Searches**

Searches will be conducted in PubMed and Web of Science for studies published between 1 January 2000 to 19 August 2020, with references of applicable studies also used to compile additional papers of interest. Additionally, the global household air pollution database (Shupler et al., 2018) was also reviewed for relevant publications.

Search terms:

Web of Science

(ALL=( "particulate matter" OR "PM 2.5" OR "PM2.5" OR pm25 OR "PM 25" ) AND ALL=( "personal exposure*" )) AND LANGUAGE: (English)

PubMed

((( "particulate matter" OR "PM 2.5" OR "PM2.5" OR pm25 OR "PM 25" ) AND ( "personal exposure*" ))) AND (("2000/01/01"[Date - Publication] : "2020/08/19"[Date - Publication]))) AND (English[Language])

Only peer-reviewed published journal articles and reports in English will be included. Conference abstracts, grey literature, letters and notes will be excluded.

**Types of study to be included**

Inclusion: Any study that has measured personal PM_2.5_ exposure through use of monitors that are worn or carried for greater than 20 continuous hours per participant throughout a typical day. Intervention studies such as changing stove type/ use of indoor filter by participants will be included.

Exclusion: Studies where no original data was analysed. Reviews or methodological papers. Studies will be excluded where participants are required to undertake activities outside their normal behaviour (i.e. requirement to take transport to fixed location each day or studies which focus on the air quality influence of specific events such as a festival). Studies which modelled or assigned personal exposure based on fixed monitors, or estimated personal exposure based on microenvironment concentrations and time-activity data were also excluded. Exposures which follow scripted activity patterns recorded by technicians to estimate a populations exposure are excluded. Studies which modelled PM_2.5_ exposure based on carbon monoxide measurements were also excluded.

**Participants/Population**

Any individual in any country going about typical daily activities. The total number of unique participants in each study needs to be 10 or greater.

**Exposure**

Mean personal PM_2.5_ exposure measurements greater than 20 continuous hours, where participants are physically required to carry a monitor.

**Comparator(s)/control**

Not relevant.

**Context**

There is no restriction on age and geographical coverage of studies.

**Main outcome(s)**

Each included study must report a summary personal PM_2.5_ exposure measurement, these can be split across different groups such as between gender, age and urban and rural locations.

**Additional outcome(s)**

Studies which report time-activity patterns, personal to ambient PM_2.5_ ratios and correlation between personal and ambient PM_2.5_ measurement will be recorded.

**Data extraction (selection and coding)**

A reviewer will screen titles and abstracts identified from the results of the systematic search and identify those that will be excluded based on the eligibility criteria. A second reviewer will review information extracted with disagreements resolved through discussion.

A standardised form will be used to extract data, including:

- Author
- Title
- Year
- Journal
- DOI
- Monitoring year
- Monitoring duration and season
- Location
- Research question
- Study design
- Participant number
  - Participant/ population characteristics (i.e. elderly, children, age, occupation, gender etc)
- Summary of findings
- Pollutant (s) measured
  - Data on PM_2.5_ exposure including but not limited to (mean, GM, median, standard deviation, GSD, range, IQR).
- Personal monitor used
- Type of personal monitor, time integrated or time resolved
- Flow rate
- Time resolution of personal monitor
- Monitor calibration and quality assurance and quality control procedures reported
- Ambient PM_2.5_ concentration
- Ambient monitor and time resolution
- Correlation between ambient and personal exposure
- Details of how activities were recorded
  - Breakdown of time activity information into home, commute, work/school, cooking/eating, other and indoor and outdoor.
- Breakdown of exposure information relating to activity
  - Subjective quality assessment (low, moderate, high) based on QA/QC of monitors, and number of participants monitored in the study.

**Quality assessment**

As this is an observational personal exposure systematic review as opposed to reviewing direct health outcomes or interventions, the subjective quality assessment will be framed around the correction and accuracy of the PM_2.5_ monitors (detection bias) to represent the populations personal exposure (selection bias). A low quality study is one that does not provide QA/QC details or the QA/QC is limited (i.e. if a time-resolved monitor is not co-located to gravimetric filter measurements or reference monitor, or when using time-integrated gravimetric monitors not following standard procedures i.e. blank filter correction, flow calibration, filters processed in a controlled temperature lab, replicate filter weighing) and participant size is small <30, a moderate quality study has either good QA/QC details or participant size is >30, while a high quality study has thorough QA/QC and representative participant size >30.

**Strategy for data synthesis**

While some simple statistical analysis will be conducted, a large proportion of the systematic review will be descriptive including discussing different sources of PM_2.5_ exposure in high, upper-middle, lower-middle and low income countries.

Where possible studies will be pooled to provide summary statistics of PM_2.5_ exposure between different income countries. Further analysis will be conducted on personal to ambient ratios and correlation of personal exposure to ambient concentrations where available.

**Analysis of subgroups or subsets**

Further quantitative and qualitative analysis will be conducted for personal PM_2.5_ exposures across gender, age and urban and rural areas where this information is available.

This protocol was not registered as PROSPERO only accepts systematic review registrations with a health-related outcome.

1. **Validation of geometric mean to arithmetic mean calculations**

Studies which provided geometric mean and standard deviations were converted to arithmetic mean so that studies summary statistics could be fairly compared using equations in Higgins et al., (2008). The equation was:

$${AM}_{c} =\exp\left( \ln\left( GM \right)+\frac{{\ln\left( GSD \right)}^{2}}{2} \right)$$

Where AM_c_ is the calculated arithmetic mean, GM is the geometric mean and GSD is the geometric standard deviation reported by the study. To test for accuracy, 11 studies (Adgate et al., 2003; Brauer et al., 2000; Brown et al., 2009; Ebelt et al., 2000; Fan et al., 2020; Minguillón et al., 2012; Nethery et al., 2008, 2012; Noullett et al., 2010; Orakij et al., 2017; Pant et al., 2017) which presented both the geometric and arithmetic means were used to calculate the difference between reported and calculated arithmetic means. The overall mean difference between calculated and reported arithmetic means was 0.21 µg/m^3^, so this conversion was deemed accurate enough to be used for the studies which did not provide arithmetic mean details.

1. **Summary of personal PM_2.5_ exposure measurements from reviewed studies split by different groups**

Table S3: Summary of measurements split across different age groups.

|  | **Number of groups measured** | | | |
| --- | --- | --- | --- | --- |
| **Age group** | **HIC** | **UMIC** | **LMIC & LIC** | **Total** |
| Adult | 75 | 72 | 26 | **173** |
| Children and infants | 25 | 6 | 15 | **46** |
| Elderly | 28 | 13 | 2 | **43** |
| Adult and child | 5 | 2 | - | **7** |
| Elderly and child | 4 | - | - | **4** |
| **Total** | **137** | **93** | **43** | **273** |
|  |  |  |  |  |

HIC: High income country, UMIC: Upper-middle income country, LMIC: Lower-middle income country, LIC: Low income country as defined by World Bank (2020).

Table S4: Summary of measurements split across different population groups

|  | **Number of groups measured** | | | |
| --- | --- | --- | --- | --- |
| **Population groups** | **HIC** | **UMIC** | **LMIC & LIC** | **Total** |
| General adult population | 51 | 27 | 9 | **87** |
| Home worker | 0 | 36 | 16 | **52** |
| School children | 24 | 6 | 10 | **40** |
| Elderly | 17 | 13 | 2 | **32** |
| Individuals with medical conditions | 21 | 1 | 0 | **22** |
| University students | 8 | 6 | 0 | **14** |
| Pregnant | 7 | 3 | 1 | **11** |
| Infant | 1 | 0 | 5 | **6** |
| School children and adult | 5 | 1 | 0 | **6** |
| School children and elderly | 3 | 0 | 0 | **3** |
| **Total** | **137** | **93** | **43** | **273** |
|  |  |  |  |  |

HIC: High income country, UMIC: Upper-middle income country, LMIC: Lower-middle income country, LIC: Low income country as defined by World Bank (2020).

1. **Summary statistics of personal PM_2.5_ exposure split by different groups**

Table S5: Summary statistics of personal PM_2.5_ exposure measurements by country income status split by urban and rural locations.

|  |  | **Number of groups measured** | **Personal PM_2.5_ exposure (μg/m^3^)** | | | | |
| --- | --- | --- | --- | --- | --- | --- | --- |
| **Income status** | **Location** |  | **Minimum** | **1^st^ Quartile** | **Median** | **3^rd^ Quartile** | **Maximum** |
| HIC | Urban | 122 | 4.3 | 11.4 | 18.6 | 26.3 | 84.1 |
|  | Rural | 15 | 7.2 | 18.3 | 20.8 | 39.3 | 88.0 |
| UMIC | Urban | 48 | 19.7 | 48.8 | 67.6 | 89.0 | 207.0 |
|  | Rural | 45 | 13.0 | 60.4 | 100.5 | 176.7 | 451.0 |
| LMIC & LIC | Urban | 11 | 36.9 | 43.8 | 53.9 | 67.8 | 484.0 |
|  | Rural | 32 | 28.4 | 61.7 | 93.9 | 185.2 | 234.3 |
|  |  |  |  |  |  |  |  |

HIC: High income country, UMIC: Upper-middle income country, LMIC: Lower-middle income country, LIC: Low income country as defined by World Bank (2020).

Table S6: Summary statistics of personal PM_2.5_ exposure divided by ambient concentrations by country income status split by urban and rural locations.

|  |  | **Number of groups measured** | **Personal/ Ambient PM_2.5_ ratio** | | | | |
| --- | --- | --- | --- | --- | --- | --- | --- |
| **Income status** | **Location** |  | **Minimum** | **1^st^ Quartile** | **Median** | **3^rd^ Quartile** | **Maximum** |
| HIC | Urban | 99 | 0.4 | 0.9 | 1.2 | 1.8 | 3.3 |
|  | Rural | 12 | 0.7 | 1.0 | 1.2 | 1.7 | 4.0 |
|  | **All** | **111** | **0.4** | **0.9** | **1.2** | **1.8** | **4.0** |
| UMIC | Urban | 26 | 0.6 | 0.9 | 1.1 | 1.4 | 4.2 |
|  | Rural | 17 | 1.4 | 1.8 | 5.1 | 8.0 | 14.6 |
|  | **All** | **43** | **0.6** | **1.0** | **1.4** | **4.1** | **14.6** |
| LMIC & LIC | Urban | 8 | 0.7 | 1.1 | 1.3 | 1.4 | 1.9 |
|  | Rural | 5 | 1.5 | 1.7 | 1.7 | 2.4 | 6.4 |
|  | **All** | **13** | **0.7** | **1.2** | **1.5** | **1.7** | **6.4** |
|  |  |  |  |  |  |  |  |

HIC: High income country, UMIC: Upper-middle income country, LMIC: Lower-middle income country, LIC: Low income country as defined by World Bank (2020).

Table S7: Summary statistics of personal PM_2.5_ exposure measurements by country income status split by age.

|  |  | **Number of groups measured** | **Personal PM_2.5_ exposure (μg/m^3^)** | | | | |
| --- | --- | --- | --- | --- | --- | --- | --- |
| **Income status** | **Age** |  | **Minimum** | **1^st^ Quartile** | **Median** | **3^rd^ Quartile** | **Maximum** |
| HIC | Child | 25 | 7.7 | 16.9 | 19.1 | 24.2 | 75.9 |
|  | Adult | 75 | 6.4 | 11.9 | 21.0 | 30.9 | 66.0 |
|  | Elderly | 28 | 4.3 | 10.9 | 14.3 | 18.6 | 26.7 |
|  | Adult and Child | 5 | 30.6 | 61.9 | 69.0 | 84.1 | 88.0 |
|  | Child and Elderly | 4 | 6.4 | 9.5 | 10.8 | 13.8 | 21.7 |
| UMIC | Child | 6 | 53.0 | 60.3 | 65.0 | 73.0 | 122.4 |
|  | Adult | 72 | 13.0 | 49.5 | 84.2 | 145.4 | 451.0 |
|  | Elderly | 13 | 47.0 | 52.0 | 65.0 | 72.5 | 135.0 |
|  | Adult and Child | 2 | 74.3 | - | 88.4 | - | 102.5 |
| LMIC & LIC | Child | 15 | 28.4 | 42.1 | 65.0 | 129.7 | 231.0 |
|  | Adult | 26 | 39.3 | 59.6 | 79.5 | 184.9 | 484.0 |
|  | Elderly | 2 | 54.0 | - | 68.1 | - | 82.1 |
|  |  |  |  |  |  |  |  |

HIC: High income country, UMIC: Upper-middle income country, LMIC: Lower-middle income country, LIC: Low income country as defined by World Bank (2020).

Table S8: Summary statistics of personal PM_2.5_ exposure measurements by country income status split by gender.

|  |  | **Number of groups measured** | **Personal PM_2.5_ exposure (μg/m^3^)** | | | | |
| --- | --- | --- | --- | --- | --- | --- | --- |
| **Income status** | **Gender** |  | **Minimum** | **1^st^ Quartile** | **Median** | **3^rd^ Quartile** | **Maximum** |
| HIC | All | 127 | 4.3 | 11.8 | 18.9 | 26.5 | 88.0 |
|  | Female | 9 | 6.4 | 11.2 | 18.5 | 29.8 | 43.8 |
|  | Male | 1 | - | - | 51.4 | - | - |
| UMIC | All | 46 | 29.3 | 53.8 | 70.1 | 98.3 | 451.0 |
|  | Female | 41 | 13.0 | 58.0 | 90.0 | 179.4 | 308.2 |
|  | Male | 6 | 19.7 | 33.9 | 60.2 | 115.4 | 133.0 |
| LMIC & LIC | All | 16 | 35.0 | 53.1 | 71.0 | 122.1 | 484.0 |
|  | Female | 22 | 39.3 | 72.2 | 118.5 | 194.1 | 234.3 |
|  | Male | 5 | 28.4 | 37.1 | 40.6 | 55.1 | 62.8 |
|  |  |  |  |  |  |  |  |

HIC: High income country, UMIC: Upper-middle income country, LMIC: Lower-middle income country, LIC: Low income country as defined by World Bank (2020).

Table S9: Summary statistics of personal PM_2.5_ exposure by country income status split by season.

|  |  | **Number of groups measured** | **Personal/ Ambient PM_2.5_ ratio** | | | | |
| --- | --- | --- | --- | --- | --- | --- | --- |
| **Income status** | **Season** |  | **Minimum** | **1^st^ Quartile** | **Median** | **3^rd^ Quartile** | **Maximum** |
| HIC | All seasons | 68 | 4.3 | 10.7 | 17.8 | 28.0 | 84.1 |
|  | Spring | 2 | 16.9 | - | 23.9 | - | 30.8 |
|  | Summer | 30 | 10.0 | 13.9 | 20.9 | 24.9 | 88 |
|  | Autumn | 11 | 8.5 | 17.2 | 20.1 | 20.3 | 31.8 |
|  | Winter | 24 | 6.3 | 14.0 | 18.5 | 35.2 | 75.9 |
|  | Not reported | 2 | 40.7 | - | 40.8 | - | 40.8 |
| UMIC | All seasons | 28 | 19.7 | 47.8 | 65.1 | 105.3 | 266.0 |
|  | Spring | 5 | 58.0 | 62.0 | 68.0 | 82.0 | 145.0 |
|  | Summer | 18 | 35.0 | 55.3 | 66.8 | 82.9 | 133.0 |
|  | Autumn | 4 | 72.5 | 80.8 | 87.7 | 94.6 | 102.5 |
|  | Winter | 22 | 35.9 | 66.6 | 128.7 | 204.7 | 451.0 |
|  | Dry | 7 | 35.5 | 90.2 | 104.0 | 125.0 | 129.0 |
|  | Wet | 6 | 13.0 | 22.3 | 26.2 | 29.8 | 34.0 |
|  | Not reported | 3 | 110.5 | 135.3 | 160.0 | 200.0 | 240.0 |
| LMIC & LIC | All | 17 | 36.9 | 55.1 | 65.0 | 144.0 | 231.0 |
|  | Summer | 1 | - | - | 53.9 | - | - |
|  | Winter | 1 | - | - | 484.0 | - | - |
|  | Dry | 11 | 35.0 | 77.0 | 104.0 | 127.5 | 210.0 |
|  | Wet | 13 | 28.4 | 40.7 | 82.1 | 127.3 | 234.3 |
|  |  |  |  |  |  |  |  |

HIC: High income country, UMIC: Upper-middle income country, LMIC: Lower-middle income country, LIC: Low income country as defined by World Bank (2020).

**References**

Adgate, J. L., Ramachandran, G., Pratt, G. C., Waller, L. A., & Sexton, K. (2003). Longitudinal variability in outdoor, indoor, and personal PM2.5 exposure in healthy non-smoking adults. *Atmospheric Environment*, *37*(7), 993–1002. https://doi.org/10.1016/S1352-2310(02)00978-0

Brauer, M., Hruba, F., Mihalikova, E., Fabianova, E., Miskovic, P., Plzikova, A., Lendacka, M., Vandenberg, J., & Cullen, A. (2000). Personal exposure to particles in Banska Bystrica, Slovakia. *Journal of Exposure Analysis and Environmental Epidemiology*, *10*(5), 478–487. https://doi.org/10.1038/sj.jea.7500136

Brown, K. W., Sarnat, J. A., Suh, H. H., Coull, B. A., & Koutrakis, P. (2009). Factors influencing relationships between personal and ambient concentrations of gaseous and particulate pollutants. *Science of the Total Environment*, *407*(12), 3754–3765. https://doi.org/10.1016/j.scitotenv.2009.02.016

Ebelt, S. T., Petkau, A. J., Vedal, S., Fisher, T. V., & Brauer, M. (2000). Exposure of chronic obstructive pulmonary disease patients to particulate matter: Relationships between personal and ambient air concentrations. *Journal of the Air & Waste Management Association*, *50*(7), 1081–1094. https://doi.org/10.1080/10473289.2000.10464166

Fan, Y., Han, Y., Liu, Y., Wang, Y., Chen, X., Chen, W., Liang, P., Fang, Y., Wang, J., Xue, T., Yao, Y., Li, W., Qiu, X., & Zhu, T. (2020). Biases Arising from the Use of Ambient Measurements to Represent Personal Exposure in Evaluating Inflammatory Responses to Fine Particulate Matter: Evidence from a Panel Study in Beijing, China. *Environmental Science & Technology Letters*, *7*(10), 746–752. https://doi.org/10.1021/acs.estlett.0c00478

Higgins, J. P. T., White, I. R., & Anzures-Cabrera, J. (2008). *Meta‐analysis of skewed data: Combining results reported on log‐transformed or raw scales*. 21.

Minguillón, M. C., Schembari, A., Triguero-Mas, M., de Nazelle, A., Dadvand, P., Figueras, F., Salvado, J. A., Grimalt, J. O., Nieuwenhuijsen, M., & Querol, X. (2012). Source apportionment of indoor, outdoor and personal PM2.5 exposure of pregnant women in Barcelona, Spain. *Atmospheric Environment*, *59*, 426–436. https://doi.org/10.1016/j.atmosenv.2012.04.052

Nethery, E., Teschke, K., & Brauer, M. (2008). Predicting personal exposure of pregnant women to traffic-related air pollutants. *Science of the Total Environment*, *395*(1), 11–22. https://doi.org/10.1016/j.scitotenv.2008.01.047

Nethery, E., Wheeler, A. J., Fisher, M., Sjödin, A., Li, Z., Romanoff, L. C., Foster, W., & Arbuckle, T. E. (2012). Urinary polycyclic aromatic hydrocarbons as a biomarker of exposure to PAHs in air: A pilot study among pregnant women. *Journal of Exposure Science & Environmental Epidemiology*, *22*(1), 70–81. https://doi.org/10.1038/jes.2011.32

Noullett, M., Jackson, P. L., & Brauer, M. (2010). Estimation and characterization of children’s ambient generated exposure to PM2.5 using sulphate and elemental carbon as tracers. *Atmospheric Environment*, *44*(36), 4629–4637. https://doi.org/10.1016/j.atmosenv.2010.08.004

Orakij, W., Chetiyanukornkul, T., Chuesaard, T., Kaganoi, Y., Uozaki, W., Homma, C., Boongla, Y., Tang, N., Hayakawa, K., & Toriba, A. (2017). Personal inhalation exposure to polycyclic aromatic hydrocarbons and their nitro-derivatives in rural residents in northern Thailand. *Environmental Monitoring and Assessment*, *189*(10), 510. https://doi.org/10.1007/s10661-017-6220-z

Pant, P., Habib, G., Marshall, J. D., & Peltier, R. E. (2017). PM2.5 exposure in highly polluted cities: A case study from New Delhi, India. *Environmental Research*, *156*, 167–174. https://doi.org/10.1016/j.envres.2017.03.024

Shupler, M., Godwin, W., Frostad, J., Gustafson, P., Arku, R. E., & Brauer, M. (2018). Global estimation of exposure to fine particulate matter (PM2.5) from household air pollution. *Environment International*, *120*, 354–363. https://doi.org/10.1016/j.envint.2018.08.026

World Bank. (2020). *World Bank Country and Lending Groups*. https://datahelpdesk.worldbank.org/knowledgebase/articles/906519-world-bank-country-and-lending-groups
